# Leucorrhœa and Ulceration of the Cervex Uteri during Pregnancy

**Published:** 1862-03

**Authors:** 


Leucorrhœa and Ulceration of the Cervex Uteri during Pregnancy.—In order to test the accuracy of M. Cazeaux's assertion that ulcerations of the cervix uteri are met with in seven eighths of pregnant women, M. Charrier instituted a careful examination of one hundred indiscriminately as they offered themselves to his notice. The following are the conclusions of the memoir he has prepared on the subject:—1. Leucorrhœa precedes and gives rise to ulceration of the cervix. 2. The congested condition and processes of hypertrophy taking place in the pelvic organs are the causes of this leucorrhœa. 3. At first a physiological condition, it may become morbid under the influence of a bad state of health. 4. Nearly two thirds (62 per 100) of pregnant women have leucorrhœa. 5. Nearly eight tenths (56 of 72) of these subjects of leucorrhœa have ulcers of the cervix. 6. Of the 56 women, 41 were multiparæ. 7. For the treatment we should confine our attention to the general condition, giving mild aperients and preparations of iron, remedying disorders of the digestive organs. Local treatment would frequently induce abortion.—*Boston Medical and Surgical Journal.*

